# Corticolimbic catecholamines in stress: a computational model of the appraisal of controllability

**DOI:** 10.1007/s00429-014-0727-7

**Published:** 2014-02-28

**Authors:** Vincenzo G. Fiore, Francesco Mannella, Marco Mirolli, Emanuele Claudio Latagliata, Alessandro Valzania, Simona Cabib, Raymond J. Dolan, Stefano Puglisi-Allegra, Gianluca Baldassarre

**Affiliations:** 1 Wellcome Trust Centre for Neuroimaging, Institute of Neurology, UCL, 12 Queen Square, London, WC1N 3BG UK; 2 Laboratory of Computational Embodied Neuroscience, Istituto di Scienze e Tecnologie della Cognizione, Consiglio Nazionale delle Ricerche (LOCEN-ISTC-CNR), Via San Martino della Battaglia 44, 00185 Rome, Italy; 3 Dipartimento di Psicologia and Centro Daniel Bovet, Sapienza Università di Roma, Via dei Marsi 78, 00183 Rome, Italy; 4 Fondazione Santa Lucia, IRCCS, Via Ardeatina 306, 00142 Rome, Italy

**Keywords:** Dopamine, Noradrenaline, Appraisal, Chronic stress, Animal model, Cortical control

## Abstract

Appraisal of a stressful situation and the possibility to control or avoid it is thought to involve frontal-cortical mechanisms. The precise mechanism underlying this appraisal and its translation into effective stress coping (the regulation of physiological and behavioural responses) are poorly understood. Here, we propose a computational model which involves tuning motivational arousal to the appraised stressing condition. The model provides a causal explanation of the shift from active to passive coping strategies, i.e. from a condition characterised by high motivational arousal, required to deal with a situation appraised as stressful, to a condition characterised by emotional and motivational withdrawal, required when the stressful situation is appraised as uncontrollable/unavoidable. The model is motivated by results acquired via microdialysis recordings in rats and highlights the presence of two competing circuits dominated by different areas of the ventromedial prefrontal cortex: these are shown having opposite effects on several subcortical areas, affecting dopamine outflow in the striatum, and therefore controlling motivation. We start by reviewing published data supporting structure and functioning of the neural model and present the computational model itself with its essential neural mechanisms. Finally, we show the results of a new experiment, involving the condition of repeated inescapable stress, which validate most of the model’s predictions.

## Introduction

Stressful events (*stressors*) are experiences that an organism appraises as difficult to deal with by reliance on its current repertoire of physiological, behavioural, and psychological responses. An initial appraisal is required to classify an event as stressful so to trigger effective (*active*) coping strategies. Once these are deployed, a second appraisal establishes whether the stressor is controllable/avoidable, hence sensitive to the organism’s reaction, or uncontrollable/unavoidable, thus requiring a shift towards a *passive* coping strategy aimed at conserving energy and resources (Folkman et al. 1986; Lazarus 1993; Huether et al. 1999; Ursin and Eriksen 2004; Anisman and Matheson 2005).

Converging evidence points to the frontal cortices as a key factor for the appraisal of controllability (Phan et al. 2004; Amat et al. 2005; Salomons et al. 2007; Ohira et al. 2008; Wager et al. 2008; Maier and Watkins 2010). However, the mechanisms involved in tuning behavioural and physiological stress responses are still mostly unexplored. The aim of the present paper is to propose a brain circuit that could translate stress appraisal into a motivational state sufficient for implementation of appropriate coping strategies.

Coping responses aimed at escaping, removing or controlling a condition appraised as stressful require high emotional/motivational arousal. Furthermore, if the stressor is experienced for the first time, the development of novel coping strategies requires focused, effortful and risky attempts. However, if the situation is insensitive to both previously established strategies and newly deployed ones, a rapid shift into passive coping is required in order to prevent sustained stress responses that are dangerous for the organism’s survival and well-being. Emotional/motivational withdrawal can stop physiological stress responses and terminate active coping (Cabib and Puglisi-Allegra 2012).

Mesoaccumbens dopamine (DA) is considered a key modulator of motivational arousal. High DA levels in the nucleus accumbens (NAcc) support effortful goal-seeking, whereas blockade of DA transmission in NAcc interferes with motivated behaviour (Salamone et al. 2003; Cagniard et al. 2006; Niv et al. 2007; Floresco et al. 2008). Moreover, DA transmission is involved in learning and NAcc is part of the complex circuit mediating the acquisition and control of goal-directed behaviour (Mannella et al. 2013). In stressed animals, NAcc DA levels undergo dramatic fluctuations that are controlled by catecholaminergic transmission in the ventromedial prefrontal cortex (vmPFC; Pascucci et al. 2007). Therefore, by modulating mesoaccumbens DA, vmPFC could tune the motivational state of the organism to the appraised situation.

Here, we propose a model of these processes. The paper first presents the biological bases of the model in the form of a review. This is not meant to be an exhaustive review of the neurobiological mechanisms involved in stress coping, but a selection of literature that has guided the development of a computational hypothesis explaining appraisal of controllability in terms of the neural mechanisms in both vmPFC and NAcc. Next, we introduce a system-level computational model. This model suggests the appraisal of controllability results from the interplay between two circuits dominated by different subregions in the vmPFC and supported by either cortical DA or norepinephrine (NE). This is the first integrated operational explanation of the observed phenomena and provides predictions in the form of simulations of expected catecholamine outflows, across a variety of conditions. Lastly, the paper presents new data testing the model’s core hypothesis. In particular, we establish a comparison between in vivo experiments testing the effects of repeated stress experience and relative simulated predictions. The results of these comparisons support the validity of the working hypotheses and the soundness of the approach (cf. Montague et al. 2012).

## Materials and methods

### The biology behind the model

**Fig. 1:**
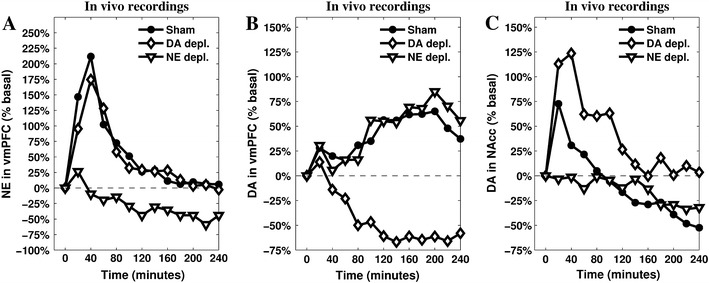
Release of NE (**a**) and DA (**b**) measured in the vmPFC, and DA measured in NAcc (**c**). Data recorded during a restraint experiment lasting 240 min and run in three different conditions: sham, depletion of vmPFC NE, and depletion of vmPFC DA. Reprinted from Pascucci et al. (2007), by permission of Oxford Univeristy Press.

The starting point in our model is a group of experiments using intracerebral microdialysis to analyse changes of catecholamine releases in vmPFC and NAcc of rats during their first experience with an uncontrollable/unavoidable stressor (Fig. 1). The results of these experiments revealed time-dependent changes of DA outflow in the NAcc and of NE and DA outflows in the vmPFC. In the first minutes following stress onset, NE in vmPFC and DA in NAcc increase in parallel, whereas DA in vmPFC shows a small and transitory peak. Blockade of NE transmission in vmPFC by selective depletion or by local infusion of an alpha1-adrenergic antagonist prevented the increase of DA outflow in the NAcc. In the course of the stress experience NE in vmPFC declines to reach basal levels, whereas DA outflow shows a second larger and sustained increase while at the same time DA in NAcc decreases below basal levels. The decrease of DA in NAcc below basal levels can be prevented by selective depletion of DA in vmPFC (Pascucci et al. 2007; Nicniocaill and Gratton 2007).

These results highlight that in stressed animals a causal relationship exists between increased NE release in vmPFC and increased DA outflow in NAcc, and between increased DA release in vmPFC and decreased DA outflow in NAcc. Converging evidences (reviewed in Cabib and Puglisi-Allegra 2012) support the view that enhanced DA outflow in NAcc is associated with expression of active coping strategies aimed at removing/avoiding the source of stress, whereas decrease of DA release below basal level is associated with expression of passive coping in unavoidable/uncontrollable stressful situation. Moreover, there is evidence supporting the view that either the large increase of DA in vmPFC or the decrease of DA in NAcc are selectively promoted by experiences appraised as uncontrollable/unavoidable (Bland et al. 2003a; Cabib and Puglisi-Allegra 1994). Therefore, the changes of catecholamine levels in the vmPFC and in NAcc that characterise the response to a novel unavoidable/uncontrollable stressor could derive from the primary appraisal of the stressfulness of a stimulus and the subsequent appraisal of its uncontrollability.

Catecholamines collected by intracerebral microdialysis derive from specific populations of projecting neurons. vmPFC NE derives from locus coeruleus (LC), part of a vast and diffuse system arising from a small population of noradrenergic cells (Glavin 1985; Aston-Jones et al. 1999; Valentino and Van Bockstaele 2001; Berridge and Waterhouse 2003). LC receives strong convergent projections from orbito-frontal cortex (OFC), anterior cingulate cortex (ACC), and central nucleus of the amygdala (Amg), a major node of the central Amg (CeA). Converging OFC and ACC inputs to LC are thought to drive transitions between phasic and tonic modes in NE neurons to fit behavioural/cognitive states with perceived environmental conditions (Aston-Jones and Cohen 2005), whereas a direct input from the CeA modulates LC neuronal activity through excitatory inputs (Van Bockstaele et al. 2001; Curtis et al. 2002; Bouret et al. 2003; Jedema and Grace 2004). Stress promotes an increase of vmPFC NE levels that exceeds those required to support cognitive functions and leads to a selective activation of alpha1 adrenergic receptors (Arnsten 2009) that indirectly stimulates DA release in NAcc (Nicniocaill and Gratton 2007).

Stress-induced changes in DA levels appear to involve mainly VTA projecting cells (Abercrombie et al. 1989; Kalivas and Duffy 1995; Barrot et al. 1999; Inglis and Moghaddam 1999; Barrot et al. 2000). These cells project toward the vmPFC as well as to the NAcc; however, these areas receive inputs from different populations of DA cells controlled by different and largely independent circuits (Carr and Sesack 2000; Margolis et al. 2006; Briand et al. 2007; Lammel et al. 2008). In particular, they receive different afferent projections from the vmPFC (Room et al. 1985; Carr and Sesack 2000; Jackson et al. 2001).

Stress-induced changes of DA levels are slow and detectable by intracerebral microdialysis (Cabib and Puglisi-Allegra 2012, for a review). This suggests that stress-induced increased DA levels depend on the removal of inhibitory constraints influencing the number of spontaneously active VTA neurons (“tonically” active neurons; Floresco et al. 2003; Grace et al. 2007) rather than on an increase in fast-spiking activity of already active neurons (phasic activity). VTA receives inhibitory inputs from the CeA which leads to an increase of NAcc DA (Ahn and Phillips 2003), suggesting that this input is part of a double inhibition mechanism (Fudge and Haber 2000; Ahn and Phillips 2002; Fudge and Emiliano 2003; Floresco et al. 2003). Therefore, CeA seems to play a major role in the promotion of an initial response to stress by corticolimbic catecholamines, in line with its involvement in emotional and behavioural stress responses (Koob 2009) and in the regulation of various neuromodulatory systems (Mirolli et al. 2010), in particular in stressful conditions (Davis and Whalen 2001).

A group of brain areas classically associated with physiological and behavioural (especially innate) responses to stressors, namely the hypothalamus, periaqueductal gray, and dorsal raphe nucleus (DR; Keay and Bandler 2001; Herman et al. 2005; Maier and Watkins 2005) are also linked to the functions of DA neurons in the VTA (Geisler et al. 2007; Rodaros et al. 2007; Omelchenko and Sesack 2010; Watabe-Uchida et al. 2012). In particular, increased serotonin (5-HT) release is correlated with increased cortical DA outflow in the condition of inescapable stress (Bland et al. 2003a), indicating that neural activity of VTA cell populations responsible for cortical DA release and DR activity (responsible for the 5-HT release) are themselves tightly correlated.

Finally, as already pointed out, frontal cortices are the major sources of both primary and secondary appraisal. The appraisal of a situation as stressful is based on the available information about the external environment and the organism’s physiological and psychological state (Folkman et al. 1986; Lazarus 1993). OFC and the ACC, involved in emotional appraisal and stress perception (Pruessner et al. 2008) can be an important source of information for CeA output, but vmPFC could play a *major* role in appraisal through the interplay between its two major components: the infralimbic (IL) and prelimbic (PL) cortices.

First, it has been demonstrated that PL constrains, whereas IL facilitates, classic physiological stress responses (Diorio et al. 1993; Sullivan and Gratton 2002; Radley et al. 2006; Tavares et al. 2009), a role also mediated by their opposing effect in controlling the activity of the DR (Radley et al. 2009). Second, results of lesion studies suggest these cortices are involved in behavioural flexibility via attentional selection (Delatour and Gisquet-Verrier 2000) and adaptation to new contingencies (Gisquet-Verrier and Delatour 2006). Moreover, PL enhances whereas IL inhibits fear reaction (Vidal-Gonzalez et al. 2006; Peters et al. 2009; Sotres-Bayon and Quirk 2010). Third, PL is involved in action-outcome learning and goal-directed behaviour expression, whereas IL is involved in switching to a stimulus–response behavioural mode (Balleine and Dickinson 1998; Coutureau and Killcross 2003; Killcross and Coutureau 2003). Finally, PL excites CeA output neurons, whereas IL inhibits them through the activation of GABAergic neurons, located in the intercalated nuclei (ITC) of the Amg (Vidal-Gonzalez et al. 2006; Peters et al. 2009).

### The computational model: core hypotheses and mechanisms

**Fig. 2:**
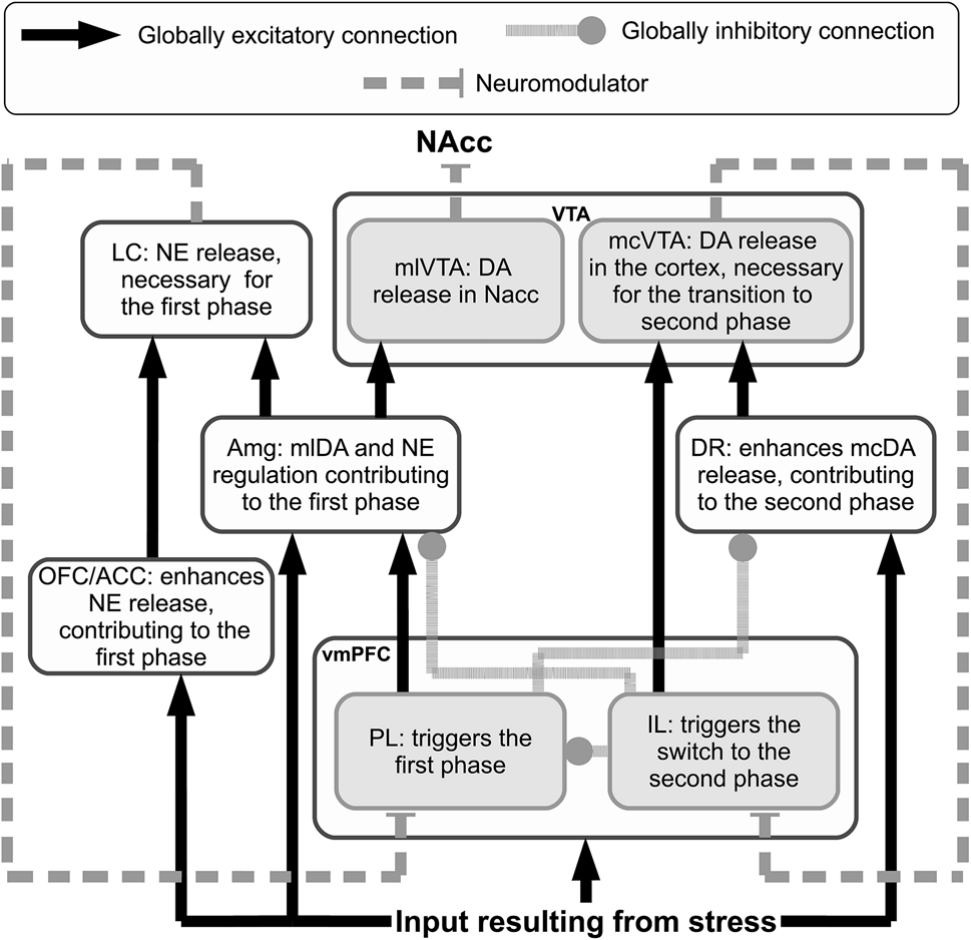
Functional representation of the architecture. This simplified representation shows the net excitatory/inhibitory influence that each component has on the target components. The *text* in the *boxes* indicates the main functional role played by each component in realising the stress responses

**Fig. 3:**
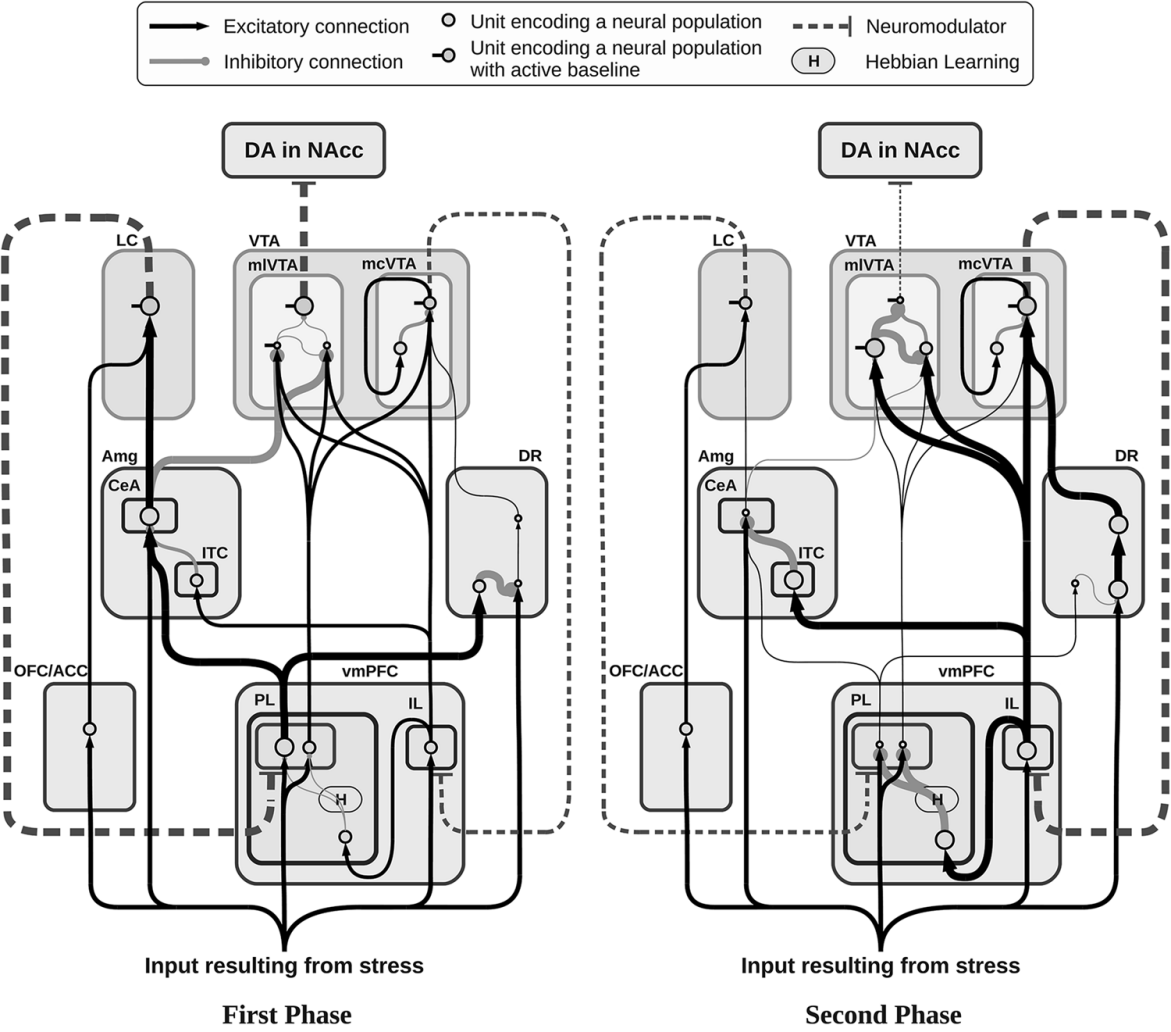
Neural architecture of the model showing its components and sub-components (*rounded square areas*), their neural assemblies (*circles*), and their connections (*links*). The size of *circles* and *links*, respectively, encode the degree of activity of neural assemblies and the strength of the signals transmitted between them. The first phase (left, active response) and second phase (right, passive response) refer to the activity recorded in the sham condition

The complex neural circuitry and mechanisms underlying an appraisal of stress controllability can be exploited to provide a causal explanation of the phenomenon itself. Here, we design a system-level model (Baldassarre et al. 2013; Fiore et al. 2014) involving a rather large number of neural systems and two neuromodulators, DA and NE: Fig. 2 shows the functional components of the model and the main relationships among them. The detailed circuitry of the model, which has been implemented in Matlab, is shown in Fig. 3.

The model relies on one pivotal hypothesis about the role played by PL and IL in uncontrollable stress conditions. In short, it is useful to distinguish three phases. First, PL-dominated circuitry, supported by NE regulation, leads active coping via the expression of goal-directed behaviour after a primary appraisal (evaluation of the presence of a stressor). Second, PL–IL interplay contributes in realising the second appraisal (evaluation of controllability of the stressor) determining the switch from the active phase to the passive one. Finally, IL-dominated circuitry, supported by cortical DA regulation, exerts control over activity of PL and various subcortical areas, causing low DA outflow in the NAcc and maintaining passive coping.

Besides the constraints deriving from the connectivity described in the previous section (see also Table 1 for full references), it is important to point out a few other features characterising the architecture and the functioning of the present model.

**Table 1:** List of key references supporting the connectivity of the model

Dopamine in NAcc	Joel and Weiner (1997)
Dopamine in vmPFC	Zhou and Hablitz (1999)
	Lewis and O’Donnell (2000)
	Tierney et al. (2008)
IL–PL interplay	Coutureau and Killcross (2003)
	Vertes (2004)
	Quirk and Mueller (2008)
	Sotres-Bayon and Quirk (2010)
vmPFC efferents to the VTA	Room et al. (1985)
	Carr and Sesack (2000)
vmPFC efferents to the DR	Vertes (2004)
	Radley et al. (2009)
vmPFC differential control over CeA and ITC in the Amg	Vertes (2004)
	Vidal-Gonzalez et al. (2006)
DR efferents to the VTA	Vertes (1991)
	Geisler et al. (2007)
	Rodaros et al. (2007)
CeA efferents to the mesolimbic VTA	Fudge and Haber (2000)
	Ahn and Phillips (2002)
	Fudge and Emiliano (2003)
OFC-ACC efferents to the LC	Aston-Jones and Cohen (2005)
CeA efferents to the LC	Curtis et al. (2002)
	Berridge and Waterhouse (2003)

The inescapable stressor is represented by a constant input signal starting 20 min after the beginning of the simulation. Information about the stressful condition has four different targets: OFC/ACC, vmPFC, CeA and DR. Consistent with described literature about neuromodulator dynamics and effects, the decoupled dynamics of vmPFC DA and NAcc DA are simulated by splitting the VTA into two separate modules, respectively, mesocortical VTA (mcVTA) and mesolimbic VTA (mlVTA). These are characterised by different afferent and efferent connectivity but share the same DA-dependent effects on their respective targets. By contrast, LC is represented by a single component causing the simulated NE release, but this neuromodulator has different effects depending on its target areas, reproducing the inhomogeneous distribution of alpha receptors (Briand et al. 2007; Arnsten 2009): the model assumes NE has an excitatory value on the vmPFC population of neurons connected to Amg and DR and an inhibitory effect on the vmPFC population of neurons connected to VTA. Finally, early simulations have driven the hypothesis that projections from DR to VTA may be asymmetrical, favouring the mcVTA module: we will discuss below how this hypothesis impacts the dynamics of the system in a relevant way.

The slow dynamics allow the use of leaky neural units (Dayan and Abbott 2001) as a building block (Baldassarre et al. 2013; Fiore et al. 2014). Therefore, each unit of the model simulates the activity of a whole neural population in a way that resembles mean field potential recordings (Bojak et al. 2003):

1 $${\begin{gathered} \tau_{j} \dot{u}_{j} = - u_{j} + b_{j} + \sum\limits_{i} {w_{ji} } a_{i} \\ a_{j} = \left[ {\tanh \left( {u_{j} } \right)} \right]^{\; + } \\ \end{gathered} }$$

where *τ*_*j*_ is the time constant, *u*_*j*_ is the action potential and *b*_*j*_ is the baseline activation of the unit *j.* $\sum\nolimits_{i} {\left[ {w_{ji} a_{i} } \right]}$ represents the sum of all products between each single input *a*_*i*_ reaching *j* and the corresponding synaptic strength *w*_*ji*_. Finally, [.]^+^ is a function returning its argument if this is positive and zero if it is negative, and tanh[.] is the hyperbolic tangent function used as a positive saturation transfer function.

The slow accumulation and reuptake of the neuromodulators in the extrasynaptic space is simulated by relying on the following equation:

2 $${\tau_{nk} \dot{l}_{nk} = - \left( {th_{nk} \;\tanh \left[ {l_{nk} } \right]} \right)\; + \;w_{nk} a_{n} }$$

Compared to the standard equation of the leaky integrator (Eq. 1), this modified version adds a reuptake capacity of the target area *k.* When the level of the neuromodulator *l*_*nk*_ drops below a threshold representing the overall reuptake capacity of the system (*th*_*nk*_), the injection of the neuromodulator *w*_*nk*_ *a*_*n*_ and its reuptake −(*th*_*nk*_tanh[*l*_*nk*_]) compensate and *l*_*nk*_ reaches an equilibrium; conversely, when it exceeds the threshold, the level of the neuromodulator starts to increase progressively (see Fellous and Linster 1998 for several ways of modelling these phenomena).

To perform the simulated depletions, we introduced a dynamic coefficient affecting the input in Eq. 2: (1 − *d*_*nk*_)(*w*_*nk*_ *a*_*n*_). When simulating the depletions of either DA or NE, the value of *d*_*nk*_ slowly grows in the range [0–1], lowering the amount of neuromodulator released. The regulation of *d*_*nk*_ towards the desired level $d^{\prime}_{nk}$ is determined by the following equation:

3 $${\tau_{{d_{nk} }} \dot{d}_{nk} = - d_{nk} + d^{\prime}_{nk} }$$

Both DA and NE activate metabotropic receptors within neurons of target areas: these receptors are involved in a range of second messenger chemical reactions. This effect is twofold: first, the metabolic status of the target neurons changes, either increasing or decreasing the chances that any incoming signal has to produce post-synaptic action potentials (i.e. the flow of ions such as Na^+^, K^+^, Ca^2+^ or Cl^−^ becomes more or less effective, depending on the activated receptor). Secondly, the presence of a neuromodulator may also result in opening new ion channels, becoming itself part of the incoming stimulus (Missale et al. 1998; Chidlow et al. 2000).

Leaky equations simulate the flow of ions as an either positive or negative numerical input for each unit, therefore neuromodulators are commonly simulated relying on *multiplicative* effects modulating the input (Fellous and Linster 1998). These effects consist in either strengthening or weakening the input generated by other neural units (multiplying or dividing it). In addition, the present model also gives an account of the opening of new ion channels caused by the presence of the neuromodulators, which are then considered as a (minor) direct input for each target unit: these are the *additive* effects. In this respect, Eq. 1 is modified as follows:

4 $${\tau_{j} \dot{u}_{j} = - u_{j} + \left( {b_{j} + \sum\limits_{i} {\left[ {w_{ji} a_{i} } \right]} } \right)\,\;\frac{{1 + \sum {\left[ {\mu_{elk} l_{k} } \right]} }}{{1 + \sum {\left[ {\mu_{dlk} l_{k} } \right]} }}\; + \;\sum {\left[ {\alpha_{elk} l_{k} } \right] - \sum {\left[ {\alpha_{dlk} l_{k} } \right]} } }$$

where the coefficients *μ*_*elk*_ and *α*_*elk*_ regulate respectively the *multiplicative excitatory* and *additive excitatory* effects of the neuromodulator *l* on target area *k*, whereas the coefficients *μ*_*dlk*_ and *α*_*dlk*_ regulate respectively the *multiplicative inhibitory* and *additive inhibitory* effects of the neuromodulator *l* on the same area. Note that the multiplicative effects depend on the size of the local glutammaergic/GABAergic signals, whereas the additive ones are independent of them.

Finally, the Hebbian learning processes leading to the increase in the strength of internal connections of vmPFC are implemented using the following learning rule:

5 $${w_{ji} \left[ t \right] = w_{ji} \left[ {t - 1} \right] + \eta \,\left[ {a_{j} - th_{j} } \right]^{ + } \left[ {a_{i} - th_{i} } \right]^{ + } }$$

where *w*_*ji*_[*t*] is the connection weight between unit *i* and unit *j* (at time *t*), *η* is a learning rate, and th_*j*_ and th_*i*_ are the thresholds that the activations of *a*_*j*_ and *a*_*i*_ have to overcome in order to trigger the learning process.

A genetic algorithm (Gulsen et al. 1995; Vander Noot and Abrahams 1998; Kapanoglu et al. 2007) is used to search the model parameters by minimising the weighted quadratic error between simulated data and target microdialyses reported in Fig. 1a–c (DA and NE dynamics in vmPFC, and DA dynamic in the NAcc, in the three different conditions reported in Pascucci et al. 2007).

### Experiments run to test the model predictions: repeated stress condition

All experiments are conducted according to the Italian national law (DL 116/92) on the use of animals for research based on the European Communities Council Directive of November 24, 1986 (86/609/EEC).

#### Animals

Male Sprague–Dawley rats (250–350 g; Charles River Labs, Calco, Como, Italy) are housed three to a cage with food and water ad libitum in animal facility where temperature is kept between 22 and 23 °C and lights is on from 7.00 a.m. to 7.00 p.m. Rats are allowed at least 1 week to acclimate to the colony room before any treatment. During this time rats are handled routinely. All surgeries and experiments are carried out between 11.00 a.m. and 6.00 p.m.

#### Drugs

Zoletil 100 Virbac, Milano, Italy (Tiletamine HCl 50 mg/ml + Zolazepam HCl 50 mg/ml) and Rompun 20 Bayer S.p.A Milano, Italy (Xilazine 20 mg/ml), purchased commercially, are used as anaesthetics, and injected i.p. in a volume of 0.5 ml/kg of each drug.

#### Microdialysis

Surgeries are performed 26–24 h before experiments. Rats are anaesthetized with Zoletil 100 and Rompun i.p. and mounted on a stereotaxic frame (David Kopf Instruments, Tujunga, CA) and implanted unilaterally with microdialysis probes in ipsilateral vmPFC and NAcc shell. Vertical concentric microdialysis probes (OD of 0, 31 mm) are prepared with AN69 fibres (Hospal Dasco, Bologna, Italy) according to the method of Di Chiara et al. (1993) as modified by Tanda et al. (1996). The probes are implanted vertically at the level of the vmPFC or the NAcc shell, according to the atlas of Paxinos and Watson (1998) (coordinates: vmPFC = A: +3.7, L: 0.9 from bregma, V: −5.0 from dura; NAcc shell = A: +1.5, L: 0.8 from bregma, V: −9.0 from dura). The length of the probes are 5 mm (membrane = 2 mm) for vmPFC and 9 mm (membrane = 2 mm) for NAcc. Each probe is fixed with epoxy glue and dental cement, and the skin is sutured. Rats are then returned to their home cages and the outlet and inlet probe tubing are protected by locally applied parafilm. The membranes are tested for in vitro recovery of DA and NE 26–24 h before the experiment. The microdialysis probe is connected to a CMA/100 pump (Carnegie Medicine, Stockholm, Sweden) through PE-20 tubing and an ultralow torque multi-channel power assist swivel (Model MCS5, Instech Laboratories, Inc., Plymouth Meeting, PA) to allow free movement. Artificial CSF (in mM: NaCl 140.0; KCl 4.0; CaCl_2_ 1.2; MgCl_2_ 1.0) is pumped through the dialysis probe at a constant flow rate of 2.1 ml/min.

Control non-stressed rats are tested in a breeding cage as stressed animals. Following the start of dialysis perfusion, rats are left undisturbed for approximately 2 h before the collection of baseline samples. The mean concentration of the three samples collected immediately before treatment (<10 % variation) is taken as basal concentration. All experimental groups are then subjected to restraint in a Plexiglas box (9 × 7 × 15 cm) provided with a sliding surface allowing rats to be gently handled during both restraining and releasing procedures (Puglisi-Allegra et al. 1991; Pascucci et al. 2007). The dialysate samples are collected every 20 min for 240 min. Placements are judged by methylene blue staining. Only data from rats with correctly placed probe are here reported. Twenty microliters of each dialysate sample is analysed by ultra-performance liquid chromatography (UPLC). The remaining 22 μl are kept for possible subsequent analysis. Concentrations (pg/20 μl) are not corrected for probe recovery.

The UPLC system consists of an Acquity UPLC (Waters Corporation, Milford, MA) apparatus coupled to an amperometric detector (model Decade II, Antec Leyden, The Netherlands) equipped by a electrochemical flow cell (VT-03, Antec Leyden) with 0.7 mm glassy carbon working electrode, mounted with a 25 mm spacer and an in situ Ag/AgCl (ISAAC) reference electrode. The electrochemical flow cell is placed immediately after a BEH C18 column (2.1 × 50 mm, 1.7 μm particle size; Waters Corporation), and set at 400 mV of potential. The column is maintained at 37 °C, the flow rate is 0.07 ml/min. The mobile phase is composed of 50 mM phosphoric acid, 8 mM KCl, 0.1 mM EDTA, 2.5 mM 1-octanesulfonic acid sodium salt 12 % MeOH and pH 6.0 adjusted with NaOH. Peak height produced by oxidation of NE and DA is compared with that produced by a standard. The detection limit of assay is 0.1 pg.

#### Experimental protocol and statistics

Experiments start 24 h after the implantation of dialysis tubes. Animals are divided into two groups (*n* = 6–7). One is subjected to four daily restraint experiences of 240 min and tested for microdialysis in 240 min restraint on the day 5, 24 h after the last stressful experience. This is compared with the second group of previously unstressed animals (controls) restrained for 240 min on day 5. Surgery is carried out 4 h after the fourth daily restraint and 24 h before restraint on day 5.

Statistical analysis are always carried out on raw data (concentrations: pg/20 μl): these are presented in figures as percent changes from baseline levels (Fig. 5).

Data on the effect of restraint on NE and DA outflow in the vmPFC and NAcc are statistically analysed by two-way ANOVAs for repeated-measure (treatment as between factor: 2 levels = control, stress, and time as within factor: 13 levels = 0, 20, 40, 60, 80, 100, 120, 140, 160, 180, 200, 220, 240 min of restraint). Simple effects are assessed by one-way ANOVA at each time point. Individual between-group comparisons are carried out, where appropriate, by post hoc test.

Restraint produces different effects on catecholamine outflow in control animals and in previously stressed animals. Statistical analysis shows a significant treatment × time interaction for NE (F12, 132 = 9.74; *p* < 0.0001) and for DA (F12, 132 = 14.71; *p* < 0.0001) in vmPFC and NAcc (F12, 132 = 8.87; *p* < 0.0001). Basal levels of prefrontal cortical amines and DA in the NAcc of control are not statistically different from repeatedly stressed rats.

## Results

### The dynamics of stress responses

As previously reported (Pascucci et al. 2007), restraint induces in control animals complex and time-dependent changes in catecholamine outflows in vmPFC and NAcc. Frontal-cortical NE shows a peak increase 20–40 min after stress onset, and then declines to reach basal levels 120 min later. Cortical DA, instead, shows a modest increase in the first 20–40 min of restraint that slowly declines before showing a much larger increase after 60–80 min from the beginning of the stress: it then remains significantly higher than basal levels for the whole duration of the experiment. In the NAcc, DA reaches a peak increase 20–40 min after stress onset then declines to reach levels significantly lower than baseline after 80–100 min of stress experience. Figure 3 illustrates the functioning of the model in simulating these changes.

The model provides a detailed hypothesis of the mechanisms behind these dynamics. In the initial phase of the experiment (Fig. 3a), putatively corresponding to the beginning of an active coping behavioural strategy, the stressor strongly activates both PL and OFC/ACC. This activity fosters high cortical NE release both directly and indirectly (via CeA), resulting in a general arousal of the system. PL also prevents DR responses to stress thus indirectly restraining cortical DA release. Eventually, the established self-feeding circuit involving PL–Amg–LC results in the constant removal of the tonic inhibitory activity of a population in the mlVTA, leading to a high efflux of DA into NAcc. Persistent input from the stressor triggers a learning process between IL and PL which strengthens the inhibition of PL output neurons. This process is assumed to correspond to the progressive inhibition of all active behaviours that fail to produce a desired outcome, i.e. in this context the removal of stress. As a result of this learning mechanism, the activity of PL output neurons slowly decreases, triggering a cascade effect that results in the transition to the second phase (Fig. 3b).

The progressive inhibition of PL affects all the nuclei in the self-feeding circuit it belongs to: first, the diminished activity reaching the CeA causes the vmPFC-NE to reach again pre-stress levels, further decreasing PL activity. Second, the now weak inhibition of DR makes this area capable of propagating its output towards the VTA, increasing cortical DA release.

**Fig. 4:**
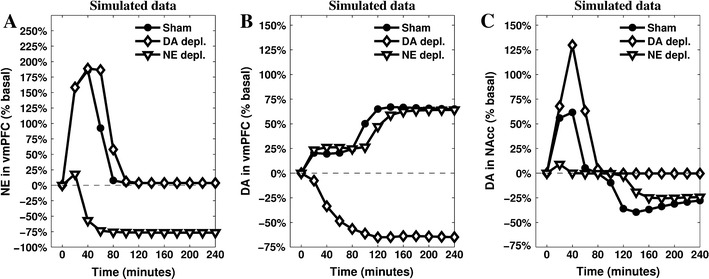
Simulations of the releases of the neuromodulators—cortical NE (**a**), cortical DA (**b**) and striatal DA (**c**)—recorded in the three conditions (sham, depletion of vmPFC NE, and depletion of vmPFC DA). The parameters have been tuned to match the original data presented in Fig. 1

The enhanced activity of IL resulting from increased cortical DA outflow speeds up the process of inhibition of both PL and CeA (via ITC). Furthermore, inputs from IL excite GABAergic populations within mlVTA, which are themselves no longer inhibited by the CeA: as a result, DA outflow in NAcc drops below the baseline. The effect of this complex circuitry on the dynamics of the neuromodulators is shown in Fig. 4, which presents the simulated outflows of the neuromodulators in the target areas.

The comparison between in vivo (Fig. 1a–c) and simulated (Fig. 4a–c) data shows a substantial match, suggesting the hypotheses the model is grounded on what may represent a sufficiently accurate explanation of the target phenomena. In particular, the model reproduces the main catecholamine dynamics in the sham condition as well as in the two conditions of either NE or DA depletion in vmPFC. Furthermore, the model exhibits a clear causal chain that furnishes a detailed account of the dynamics occurring during cortical depletions.

In the model, the vmPFC NE depletion causes a loss of about 10 % in the peak response of PL during the first phase, followed by the anticipation of its decrease of about 20 min. This diminished activity propagates to CeA, which is no longer able to overcome the “gate” created in the mlVTA by GABAergic interneuron populations. This is the main reason why NE depletion in vmPFC prevents NAcc DA from increasing during the first phase. At the same time, PL low activation slows down the IL–PL Hebbian learning process resulting in a delayed transition to the second phase.

The vmPFC DA depletion greatly diminishes the activity in IL during the whole test, slowing down IL–PL learning process and the consequent inhibition of PL activity. The stronger and more persistent activity of PL supports a higher activation of CeA and a longer accumulation of DA released in NAcc. Deprived of the excitatory effect caused by cortical DA, IL no longer shows its inhibitory effect on mlVTA, which—in the sham condition—is the cause for NAcc DA drop below baseline.

### Repeated stress condition: predictions and in vivo tests

To validate the core hypothesis of the model, we put its predictions to a test with results coming from experiments not used to tune the parameters of the model. The predictions regard the effects that a repeated experience of the restraint stressing condition, putatively causing cumulative learning within the IL–PL subsystem, might have on the outflows of the analysed catecholamines. The model relies on the hypothesis that a learning process in vmPFC is responsible for triggering a cascade effect on the subcortical areas, eventually causing the switch from active to passive coping strategies marked by the varying DA outflow in NAcc. The experiment providing the target data refers to naive rats, experiencing restraint for the first time (Pascucci et al. 2007). Thus, if the rat has already experienced restraint, it is reasonable to assume a memory of the repeated stressful experience is preserved in the vmPFC. In the model, this memory is simulated increasing the initial value of the IL–PL connection.

**Fig. 5:**
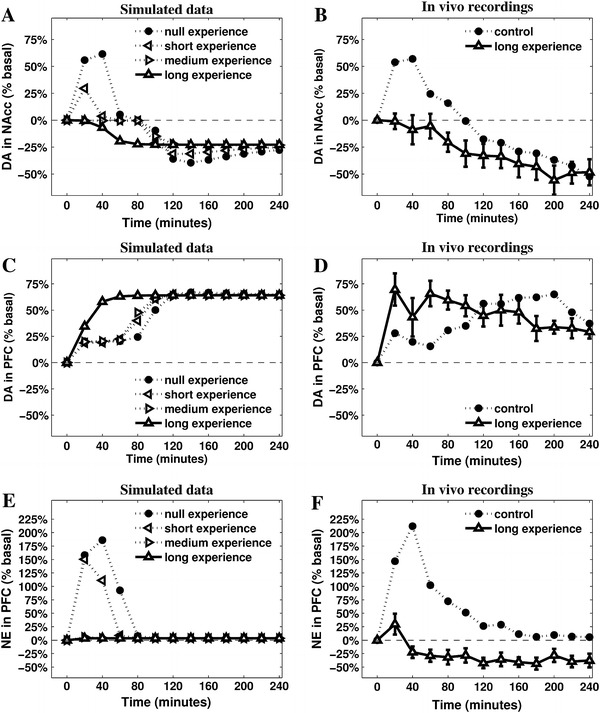
Predictions suggested by the model (**a**, **c**, **e**) and validation via in vivo data coming from new recordings of NAcc DA (**b**), vmPFC DA (**d**) and vmPFC NE (**f**) after 5 daily repetitions of mechanic restraint. The model shows a high degree of accuracy in predicting DA outflows in the NAcc (also including the medium experience, which matches data described in Imperato et al. 1992, 1993). It also manages to provide a less accurate but still valuable account of the DA release in the vmPFC, but it does not reproduce correctly the NE release in vmPFC

We set the IL–PL cortical inhibitory connection to different initial values, thus capturing the effect of different intensities of previous experiences, and assumed a partial spontaneous recovery between daily experiences. The results (Fig. 5a) show an interesting discontinuity of NAcc DA dynamics during the test when the IL–PL connection value is gradually moved from above (null/short experience) to below (long experience) a critical value of about −1 (medium experience). In particular, short experience causes a lower DA release in NAcc in the active phase, but it does not alter the timing of the passage from the active phase to the passive one. A medium experience decreases the initial DA release in NAcc to basal levels, but still leaves unaltered the timing of the passage to the second phase of coping. Finally, and notably, a long previous experience of the stressing condition causes an *anticipation* of the second phase.

**Fig. 6:**
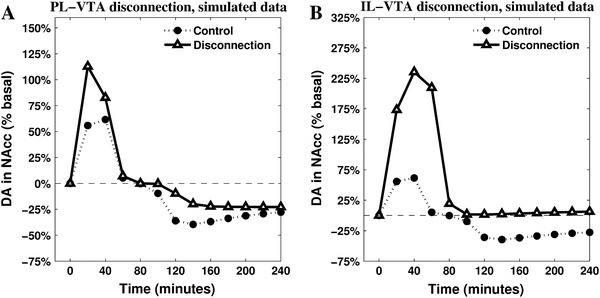
Two untested predictions produced by the model. The mesolimbic DA release is recorded after disconnecting two neural areas of the model (*blank triangle lines*) and is compared with the known data characterising sham rats (*filled circle lines*). The disconnections affect efferent projections of either PL (**a**) or IL (**b**) and their targeted areas in the VTA

Previous experiments (Imperato et al. 1992, 1993) have recorded DA release in NAcc during a restraint test lasting 120 min carried out after previous moderate exposures to the restraint stressing condition (5 repetitions of 60 min, one per day). The results show the absence of the initial peak of NAcc DA release (active coping phase) followed by a decrease below the baseline after 70–80 min (marking the beginning of passive coping). These results are consistent with the prediction of the model in relation to the moderate experience (Fig. 6a, medium experience). Since the parameters of the model were not tuned to produce this result, this is a first validation of a prediction of the model (cf. Alexander and Brown 2011).

Given the positive results of this first test, a new set of experiments were carried out to acquire new data in relation to the effects of a prolonged experience of the restraint condition. A series of five repetitions of 240-min restraint (see "Experiments run to test the model predictions: repeated stress condition" for details) has been used to produce a “long experience” stressing condition. Previously stressed animals show a slight increase of prefrontal NE outflow followed by a decrease below basal levels from 40 min throughout. DA in the vmPFC has a sudden substantial increase which is maintained through the experiment, except for a slight reduction in the last 60 min. In the NAcc, DA does not increase during the first 40–60 min, and it decreases progressively below basal levels from 60 min onwards.

Figure 5 shows the empirical data partially confirming predictions of the model. DA in NAcc is the most accurate prediction (Fig. 5b): it soon decreases below the basal level, clearly marking the anticipation of the passive coping phase. The series representing the dynamics of DA in vmPFC (Fig. 5d) can also be considered as a successful validation of the model’s predictions: the outflow increases immediately after the beginning of the stressing experience maintaining a release well above the baseline for the whole duration of the test. Finally, the model partially fails to accurately reproduce the dynamics of cortical NE outflow: as predicted, the empirical data (Fig. 5e) do not show the initial high response recorded in naive rats, but the model is unable to simulate the almost constant low release of this neuromodulator. The reason is that the architecture of the model is not conceived to simulate below the baseline NE outflows at any time (apart from the depletion condition). Assuming the model does not require the addition of further components to the brain areas it currently simulates, this decrease can be explained by a diminished input reaching LC: e.g. repeated exposure to the stressor might cause a further decrease of the activity in the Amg (consistently with data reported in mice by Gilabert-Juan et al. 2011), which would result in a more complex homoeostasis involving PL, Amg and LC.

Overall, these results are consistent with a hypothesis assuming previous experience of the same uncontrollable stressor leads to a fast appraisal of uncontrollability in the vmPFC, erasing active coping responses and causing an immediate passage to passive coping ones. Furthermore, these new data confirm the inverse correlation between cortical and limbic DA dynamics which may be well described in terms of the two competing circuits dominated by either PL or IL.

The model provides several other predictions testable in future experiments. Among these, we briefly show here the effects of two possible disconnections highlighting the core assumptions of the model in the regulation of NAcc DA. These are simulated setting the relevant connection weights to zero and leaving all other parameters of the model unaltered. The resulting dynamics are reported in Fig. 6.

The comparison between the simulated PL-VTA and IL-VTA disconnections reveals the importance of a globally inhibitory effect that vmPFC exerts on NAcc DA levels. When IL-VTA connections are removed, the simulations show a higher first response with an even more significant increase of DA in NAcc. Furthermore, this comparison allows underlying the different role played by the two cortices in causing the switch to the second phase. In particular, the IL-VTA disconnection shows a baseline DA in NAcc outflow in the second part of the simulation (Fig. 6b), entailing the absence of a passive coping phase. These dynamics, similar to the ones recorded after the cortical DA depletion (Fig. 1b), highlight the specific role played in the model by IL—and the circuitry it controls—in inhibiting NAcc DA release when its activity is sufficiently strong due to the presence of cortical DA. These predictions can be tested using combined contralateral lesions (e.g. see Coutureau et al. 2009).

## Discussion

The present model proposes a causal explanation of the neural mechanisms underlying the appraisal of controllability in the condition of long-lasting, inescapable stress. The core hypothesis assumes the learning process taking place in vmPFC is responsible for inhibition of overall activity expressed by a system pivoting on PL, Amg and cortical NE, in favour of a system involving IL, DR and cortical DA. The balance between these two systems controls the outflow of NAcc DA and therefore the motivational state and the stress coping strategy employed by the agent: high DA outflow in the NAcc drives active responses to attempt escaping (Salamone et al. 2007; Niv et al. 2007) and low DA outflow in NAcc drives passive responses and decreased overt activity (Ventura et al. 2002; Baldo and Kelley 2007; Phillips et al. 2007).

This hypothesis is consistent both with a general role ascribed to vmPFC as a key for control of hormonal and behavioural stress responses (Cabib et al. 2002; Scornaiencki et al. 2009; Maier and Watkins 2010) and with recorded data related to the catecholamine regulation of this neural region. Indeed, high cortical NE release is correlated with general arousal, as required in the face of an unknown stressful situation (Aston-Jones et al. 1999; Berridge and Waterhouse 2003), and slightly above-baseline cortical DA outflow plays an important adaptive role by preventing excessive behavioural and physiological stress reactivity (Sullivan 2004). The model assumes that a learning process in vmPFC is activated by IL due to the persistence of the stressor: IL detects the failure of the active coping attempts and progressively increases a constant inhibitory effect on PL-dominated circuitry. This is consistent with the view stating that learning processes lead to active inhibition, rather than forgetting, of those behaviours that are no longer adaptive (Quirk 2002). Several studies support the hypothesis that IL plays a key role in these inhibitory processes in its complex interplay with PL (Rhodes and Killcross 2004; Lebrón et al. 2004; Radley et al. 2006, 2008; Van Aerde et al. 2008), while the learning process assumed in the model is consistent with recorded hypertrophy of dendrites of the vmPFC interneurons induced by inescapable stress (Gilabert-Juan et al. 2012). The subsequent shift to passive coping strategies is strengthened by the readjustment of the catecholamine outflows. First, NE in vmPFC returns to pre-stress levels, thus diminishing arousal, and second, high DA release in PFC favours processing internal information rather than external stimuli, strengthening cognitive perseverance and internal focus (Cohen et al. 2002).

The tuning of the model’s parameters has been carried out to match a set of data described in previously published experiments (Pascucci et al. 2007). On this basis the model provides a number of predictions: here we focus on those putting to a test the core hypothesis concerning the role of the vmPFC and the competition of the two described circuitries in controlling the DA outflow in NAcc. The condition of repeated restraint is particularly useful in allowing the model to simulate complex dynamics. This condition is obtained via a manipulation of the initial strength of the IL–PL inhibitory connection, considered as proportional to the amount of a residual learning (a memory) caused by the length of the agent’s previous experience with the stressing stimulus. Increasing the initial value of this connection produces an interesting discontinuous effect on NAcc DA dynamics, showing the anticipation of the second–passive–phase (marked by below-baseline NAcc DA) only occurs after the initial active response has been completely erased (Fig. 5a). The prediction about a short experience of stressing condition is confirmed by previously published experiments (Imperato et al. 1992, 1993), whereas the new experiment here reported validates the prediction concerning a long-lasting experience of the same stressor. A fair degree of accuracy also characterises the predicted dynamics of cortical DA release after long experience. It is important to highlight the model causally correlates high activity in IL-dominated circuitry (involving cortical DA) with the below-baseline release of DA in NAcc: the timing reported in the new in vivo recordings (Fig. 5b, d) are consistent with this hypothesis.

Despite the fact the model does not simulate correctly the dynamics of cortical NE release in the tested condition of repeated stress (Fig. 5e, f), these data do not falsify the model’s causal explanation of the appraisal of controllability. Indeed, even if a more comprehensive model may be required to address these new dynamics, the core hypothesis about the two competing circuitries dominated by either PL or IL is consistent with the recorded lack of NE response.

It is also interesting to point out that the implementation of the model has driven a specific hypothesis concerning the DR and its supposed asymmetrical activation of the DAergic neurons in the VTA. There is good evidence regarding connections from DR to VTA as a whole (Geisler et al. 2007; Rodaros et al. 2007; Omelchenko and Sesack 2010; Watabe-Uchida et al. 2012), but little empirical evidence in support of the fine-grained asymmetry required by the model. To shed more light on this issue it would be interesting to measure the outflow of cortical 5-HT during restraint: the model predicts 5-HT increases during the passive coping phase with a timing consistent with the highest increase characterising cortical DA release. This correlation, which has been already recorded in a different stressing condition (Bland et al. 2003a), would also support the existence of a relation between the mechanisms underlying stress coping and those responsible for learned helplessness (Maier and Watkins 2005; Amat et al. 2005; Maier and Watkins 2010). When considering this set of studies, it must be noticed that the classic yoked-shocks paradigm report conflicting results when considering the mesoaccumbens DA response to controllable and uncontrollable stress. A 1994 study reports that mice controlling shock delivery show high levels of the extracellular DA metabolite 3-methoxytyramine (3-MT), whereas their yoked counterparts show levels of 3-MT significantly lower than those of unhandled controls (Cabib and Puglisi-Allegra 1994). In contrast, a study using intracerebral microdialysis reported that both shocked and yoked rats show only a temporary increase of mesoaccumbens DA outflow (Bland et al. 2003b). Differences in the species or the method used to measure extracellular DA (DA available for transmission) cannot account for these different findings because time-dependent fluctuations of NAc 3-MT tissue levels (early increase followed by decrease below basal levels) promoted by exposure to restraint or uncontrollable shock in mice are identical to the fluctuations of NAcc DA outflow measured by microdialysis in restrained rats (Puglisi-Allegra et al. 1991). Instead, the lack of similar fluctuations of NAcc DA outflow in yoked rats is well explained by different duration and quality of the stress experience. It should be pointed out that the procedure used in the rat experiment (Bland et al. 2003b) includes a progressive increase of the response requirement and of shock intensity. This test was not intended to influence the behavioural response adopted by animals facing controllable or incontrollable stress, but to promote reliable and lasting learned helplessness which requires removal of inhibition on DR 5-HT neurons by the vmPFC (Amat et al. 2005). Instead, as already discussed, coping expressed by animals exposed for the first time to unavoidable/uncontrollable stressors is dependent on NAcc DA transmission (see Cabib and Puglisi-Allegra 2012 for review). In line with this hypothesis, temporary inactivation of the vmPFC, by focal administration of the GABA agonist muscimol, does not reduce expression of active coping in rats exposed to an escapable shock but promotes expression of learned helplessness by these animals 24 h later (Amat et al. 2005).

The proposed model opens paths for further investigations. From a computational perspective, it will be useful to investigate the interactions existing between multiple neuromodulators targeting the same areas, in particular the PFC (Briand et al. 2007) so as to remove the independence between them assumed here. From a neurocognitive perspective, the model’s hypotheses regarding the putative functional roles of the various components, neuromodulators, and processes pose interesting questions. Among these, the effects of NE and DA on goal-directed behaviour and the progressive inhibition IL exerts on PL entail an action-failure detection mechanism within IL (Mannella et al. 2013). Eventually, a better understanding of IL–PL interplay and the causal mechanisms realising the appraisal of controllability may provide a fruitful framework for translational studies of disorders related to post-traumatic stress and chronic depression (cf. Milad and Quirk 2012), also considering functional homologies (Alexander and Brown 2010, 2011) and converging data recorded in primates (Drevets et al. 2008).
